# Exploring the genotypic and phenotypic differences distinguishing *Lactobacillus jensenii* and *Lactobacillus mulieris*

**DOI:** 10.1128/msphere.00562-22

**Published:** 2023-06-27

**Authors:** Adriana Ene, Swarnali Banerjee, Alan J. Wolfe, Catherine Putonti

**Affiliations:** 1 Bioinformatics Program, Loyola University Chicago, Chicago, Illinois, USA; 2 Department of Mathematics and Statistics, Loyola University Chicago, Chicago, Illinois, USA; 3 Department of Microbiology and Immunology, Stritch School of Medicine, Loyola University Chicago, Maywood, Illinois, USA; 4 Department of Biology, Loyola University Chicago, Chicago, Illinois, USA; University of California, Davis, Davis, California, USA

**Keywords:** *Lactobacillus jensenii*, *Lactobacillus mulieris*, urogenital microbiome, urinary tract, urinary microbiome

## Abstract

*Lactobacillus crispatus*, *Lactobacillus gasseri*, *Lactobacillus iners*, and *Lactobacillus jensenii* are dominant species of the urogenital microbiota. Prior studies suggest that these *Lactobacillus* species play a significant role in the urobiome of healthy females. In our prior genomic analysis of all publicly available *L. jensenii* and *Lactobacillus mulieris* genomes at the time (*n* = 43), we identified genes unique to these two closely related species. This motivated our further exploration here into their genotypic differences as well as into their phenotypic differences. First, we expanded genome sequence representatives of both species to 61 strains, including publicly available strains and nine new strains sequenced here. Genomic analyses conducted include phylogenetics of the core genome as well as biosynthetic gene cluster analysis and metabolic pathway analyses. Urinary strains of both species were assayed for their ability to utilize four simple carbohydrates. We found that *L. jensenii* strains can efficiently catabolize maltose, trehalose, and glucose, but not ribose, and *L. mulieris* strains can utilize maltose and glucose, but not trehalose and ribose. Metabolic pathway analysis clearly shows the lack of *treB* within *L. mulieris* strains, indicative of its inability to catabolize external sources of trehalose. While genotypic and phenotypic observations provide insight into the differences between these two species, we did not find any association with urinary symptom status. Through this genomic and phenotypic investigation, we identify markers that can be leveraged to clearly distinguish these two species in investigations of the female urogenital microbiota.

**IMPORTANCE:**

We have expanded upon our prior genomic analysis of *L. jensenii* and *L. mulieris* strains, including nine new genome sequences. Our bioinformatic analysis finds that *L. jensenii* and *L. mulieris* cannot be distinguished by short-read 16S rRNA gene sequencing alone. Thus, to discriminate between these two species, future studies of the female urogenital microbiome should employ metagenomic sequencing and/or sequence species-specific genes, such as those identified here. Our bioinformatic examination also confirmed our prior observations of differences between the two species related to genes associated with carbohydrate utilization, which we tested here. We found that the transport and utilization of trehalose are key distinguishing traits of *L. jensenii*, which is further supported by our metabolic pathway analysis. In contrast with other urinary *Lactobacillus* species, we did not find strong evidence for either species, nor particular genotypes, to be associated with lower urinary tract symptoms (or the lack thereof).

## INTRODUCTION

Lactobacilli are common colonizers of the human microbiome, including the gastrointestinal (GI) tract, urinary tract, and vagina (1 - 3). *Lactobacillus* species, namely *Lactobacillus crispatus*, *Lactobacillus gasseri*, and *Lactobacillus jensenii*, are considered core members of the urinary microbiota (urobiome) of females with and without urinary tract symptoms (3 - 10). They are posited to be regulators of these communities. Generally, Lactobacilli create an acidic environment that restricts the growth of pathogens (11). Individual species can also play key roles inhibiting or killing uropathogens (12, 13). For instance, prior research has shown that *L. crispatus* provides protection against uropathogenic *Escherichia coli* (14). Furthermore, *L. gasseri*, *Lactobacillus iners*, *Lactobacillus paragasseri*, and *L. jensenii* are all associated with probiotic capabilities (12, 15, 16), which may explain previous observations of *Lactobacillus*-dominated urobiomes for asymptomatic females (8, 17).

Prior studies have found *L. jensenii* in the urobiome of both symptomatic and asymptomatic females (8, 17 - 24). In a recent study, *L. jensenii* was found to be one of the most frequently detected species within the urobiomes of females with stress urinary incontinence symptoms (21). *L. jensenii* also has been routinely reported in the vaginal microbiome (10, 25 - 30). Detection of *L. jensenii* in these studies often relied on 16S rRNA gene sequencing of the V4 region in urobiome studies or the V1–V3 or V3–V4 regions in vaginal microbiome studies (10, 19, 25 - 30). Recently, a new *Lactobacillus* species, *Lactobacillus mulieris*, was discovered and characterized (31). The 16S rRNA gene sequence of this new species is nearly identical to that of *L. jensenii*, distinguishable by just two nucleotide differences, neither of which occurs within the commonly targeted hypervariable regions (32). Thus, previous associations of *L. jensenii* and symptoms (or the lack thereof) in the urogenital tract are incomplete as they were conducted prior to the characterization of *L. mulieris* or based on the 16S rRNA gene sequence only.

Given its putative important role in the urobiome, here we present further characterization of *L. jensenii* and *L. mulieris* from the urinary tract. Building upon our prior genomic analysis of *L. jensenii* and *L. mulieris* genomes (32), 61 genome assemblies, including nine new strains sequenced here, were examined. Forty-one strains in this analysis were isolated from the urinary tract, most from our own collection. Through our analysis of the core genome, 16S rRNA gene sequence, and biosynthetic gene clusters (BGCs), we could clearly distinguish between the two species while also identifying a genomospecies of *L. mulieris*. Complementing this bioinformatic analysis, we conducted phenotypic characterization of 37 urinary strains representative of both *L. jensenii* and *L. mulieris*. Here, we show that the two closely related species utilize different carbohydrates, an observation that can be supported by metabolic pathway analysis. Collectively, we have identified genotypic and phenotypic markers to distinguish between *L. jensenii* and *L. mulieris* in studies of the female urogenital microbiota.

## RESULTS AND DISCUSSION

### Genotypic differences between *L. jensenii* and *L. mulieris*

Our genomic analysis of *L. jensenii* and *L. mulieris* included 61 genomes of strains isolated from urinary, vaginal, and fecal samples (Table S1). The majority (*n* = 41) were isolated from the urinary microbiota, including nine new strains sequenced as part of this effort and deposited in GenBank. The 16S rRNA gene sequences from the 61 genomes were examined. Seven of the genomes, however, did not include a full 16S rRNA gene sequence: UMB3442, UMB0847, 269-3, IM1, IM3, UMB9245, and UMB7, and thus were excluded from this 16S rRNA gene sequence analysis. When multiple 16S rRNA gene sequences were identified, all copies were included in the set of sequences. The resulting phylogeny shows a clear distinction between the 16S rRNA gene sequences from *L. jensenii* and *L. mulieris* strains (Fig. S1). For genomes with more than one 16S rRNA gene sequence, the intragenomic variation was lower than the interspecies variation. While variation exists between the 16S rRNA gene sequences of the same species, only two nucleotide positions can unambiguously distinguish *L. jensenii* strains from *L. mulieris* strains: position 76 (T/A) and 399 (C/A).

**Fig 1:**
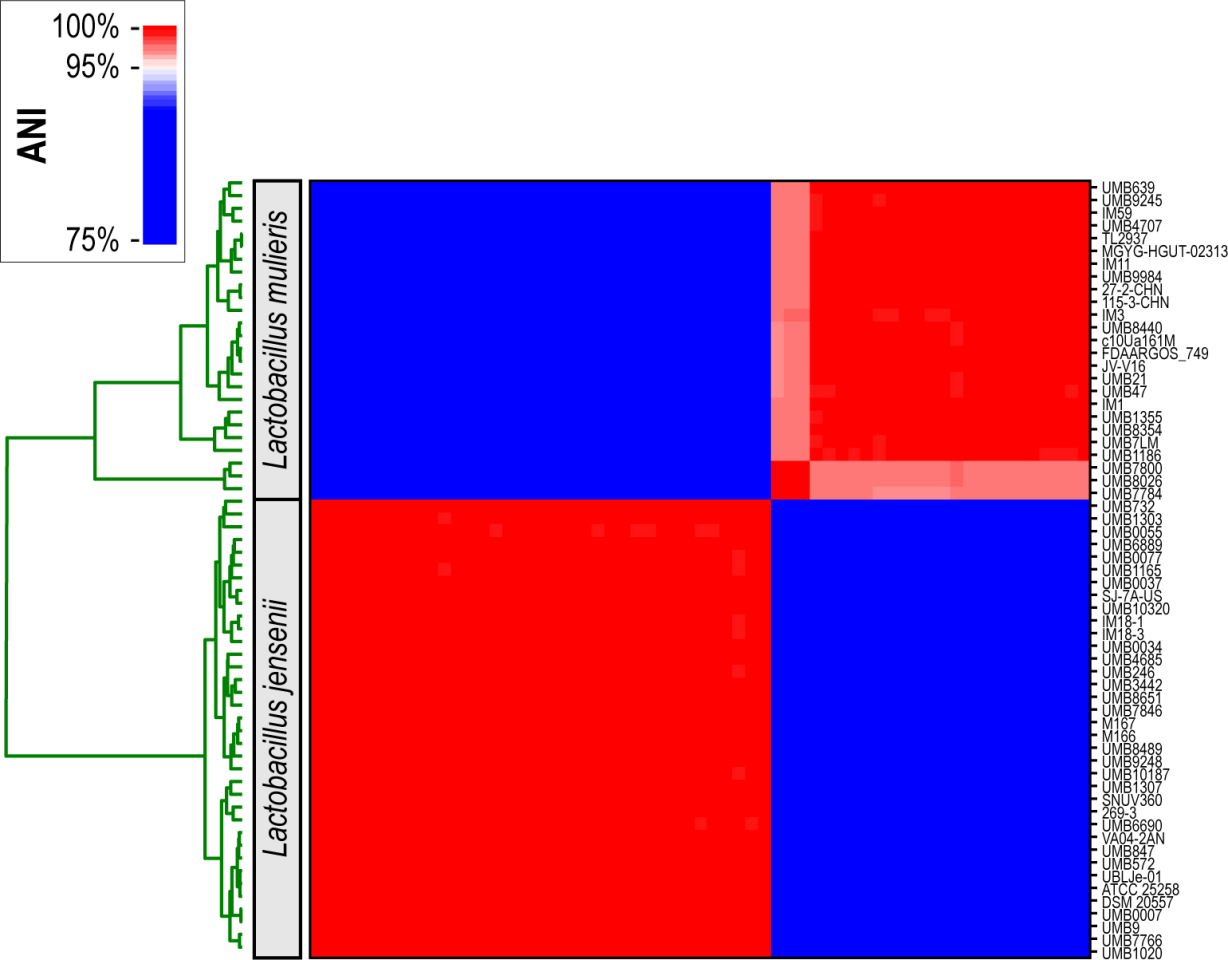
ANI analysis of *L. jensenii* and *L. mulieris* strains. The top rectangle on the upper left indicates the *L. mulieris* strains, while the bottom rectangle below it indicates the *L. jensenii* strains. The tree shown in green indicates the cladding structure of the strains based upon the pairwise ANI values. ANI, average nucleotide identity.

Next, whole genome sequences were examined. Pairwise average nucleotide identity (ANI) values ranged between 88.66% and 100%. Using the commonly used 95% ANI threshold for species designation (33), strains of *L. jensenii* and *L. mulieris* can be distinguished (Fig. 1). In total, 36 strains were identified as *L. jensenii* and 25 strains were identified as *L. mulieris*. Henceforth, the strains are identified by the species indicated from this ANI analysis. The average pairwise ANI between the *L. jensenii* and *L. mulieris* strains is 88.96%, ranging between 88.66% and 89.77% (Table S2). The average pairwise ANI between *L. jensenii* strains is 99.85%, while the average pairwise ANI of *L. mulieris* strains is 99.27%.

As shown in Fig. 1, there are three genomes within the *L. mulieris* group that form their own subgroup: UMB7784, UMB7800, and UMB8026. Their pairwise ANIs compared with the *L. mulieris* type strain c10Ua161M^T^ (GCA_007095465) are 97.17%, 97.35%, and 97.34%, respectively (31). When examining the tree within the ANI heatmap (Fig. 1, in green), one can see that the three strains clade distinctly from the remaining *L. mulieris* strains. Previously, we detected the ANI difference between UMB7784 and other *L. mulieris* strains (32); at that time, UMB7800 and UMB8026 were not sequenced. Because these three strains do not meet the conventional 95% threshold (33), we have assigned them to the *L. mulieris* species. These three divergent *L. mulieris* strains came from our collection and were isolated from voided urine samples from three different females. Interestingly, all three females were clinically diagnosed with a recurrent urinary tract infection (rUTI) (Table S1). It is worth noting, however, that another *L. mulieris* strain isolated from a catheterized urine sample from a female with rUTI (strain UMB9245) is not included in this clade; likewise, *L. jensenii* strains were isolated from both voided and catheterized urine samples from females with rUTI (Table S1). We hypothesize that these three genomes may represent an emerging genomospecies.

**Fig 2:**
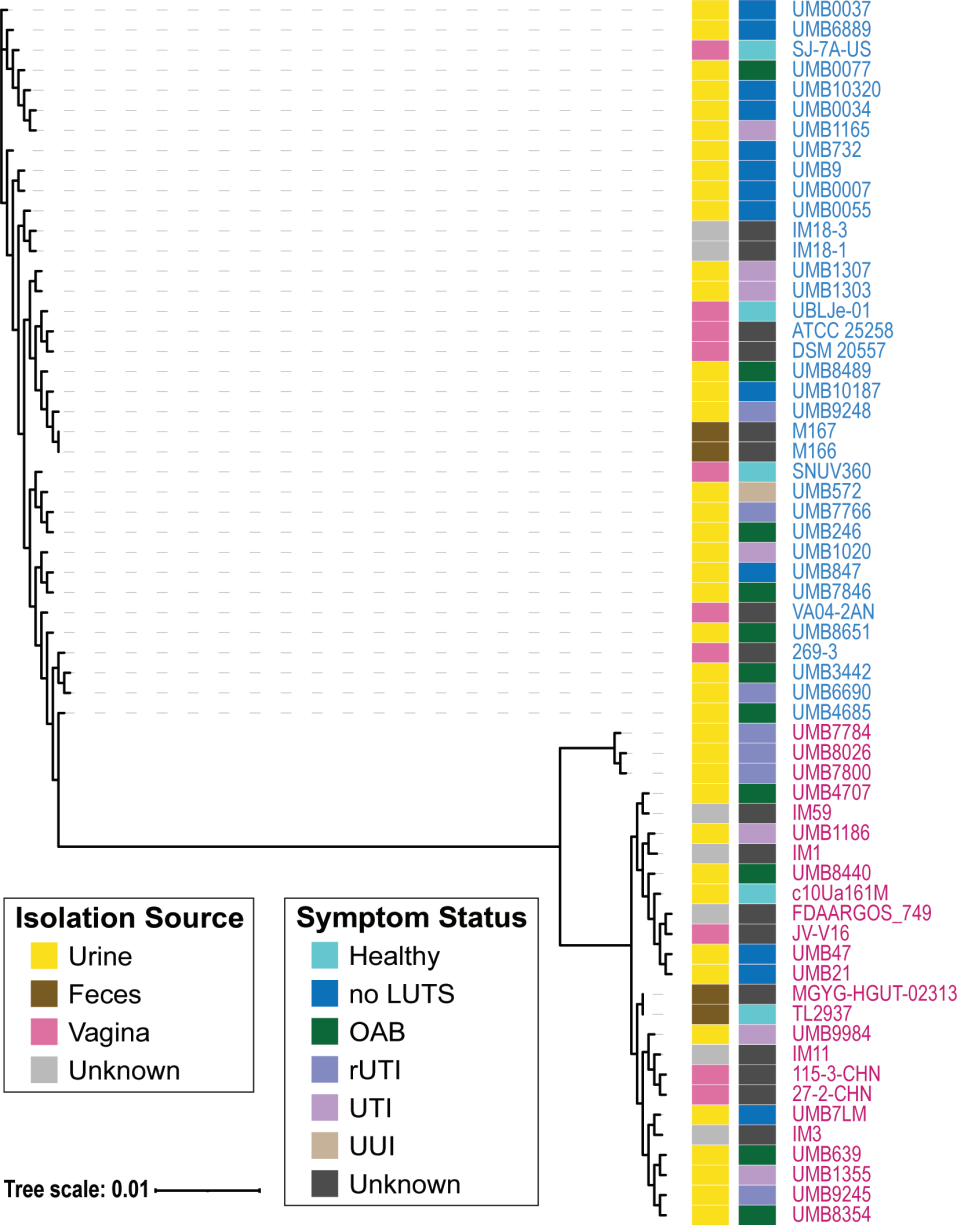
Core phylogenetic tree of *L. jensenii* (text in blue) and *L. mulieris* (text in pink) with the isolation source of the strains labeled by the first color strip and the symptom status of the individuals from which the strains were isolated labeled by the second color strip.

Next, we identified the pangenome and the set of conserved single-copy core genes of the 61 *L*. *jensenii* and *L. mulieris* genomes. The pangenome includes 2,636 unique genes. The single-copy core genome consists of 589 genes. (Henceforth, the single-copy core will be referred to as the “core genome,” indicative of the presence of the gene in all genomes examined.) The amino acid sequences of the core genome were aligned, and a phylogenetic tree was derived (Fig. 2). This tree shows the distinct *L. jensenii* and *L. mulieris* clades. Thus, the core genome is a sufficient signal of the divergence of the two species. This tree further confirms our findings from the ANI analysis (Fig. 1), as well as our prior core genome analysis of a subset of these genomes (32). It is important to note that both species include strains isolated from urine, fecal, and vaginal samples (Fig. 2). Furthermore, both species include urinary isolates from females with and without lower urinary tract symptoms. Thus, neither species appears to be niche-specific or urinary symptom-specific.

From this tree, we can also see that the three genomes within the subclade of *L. mulieris* based on ANI (Fig. 1) also constitute a subclade based upon their core genome sequences. Thus, the divergence between the primary clade of *L. mulieris* and the subclade of UMB7784, UMB7800, and UMB8026 is not simply from the acquisition of genes. Core gene nucleotide sequences have accumulated mutations that distinguish these two groups of *L. mulieris*. As previously mentioned, this subclade does not meet the ANI threshold commonly used for distinguishing species. It may represent an emerging species. Future isolation of strains belonging to this subclade and *L. mulieris* is needed to ascertain if this subclade is in fact a new species.

The differences between *L. jensenii* and *L. mulieris* also can be seen in the functional capacity of their encoded genes. Using the Clusters of Orthologous Groups of proteins (COGs) database (34), gene functionality unique to each species was identified (Tables S3 and S4). Most notably, *L. mulieris* strains encode different functionalities related to carbohydrate catabolism and transport. The carbohydrate pathways are essential in surviving competitive environments such as the bladder, where pathogenic species like *E. coli* can thrive (35). For example, *L. mulieris* encodes for COG1363, alpha-glucosidase/xylosidase, which can catalyze the transfer of alpha-xylosyl residue from alpha-xyloside to xylose, glucose, maltose, nigerose, sucrose, and trehalose. This might be indicative of *L. mulieris* being able to utilize different carbohydrates, the impetus for our empirical work described later.

### Identifying BGCs

Given antimicrobial properties that have been historically associated with *L. jensenii*, we next examined the 61 genomes for the presence of biosynthetic gene clusters (BGCs). Three different classes of BGCs were identified: ribosomally synthesized and post-translationally modified peptide (RiPP) products , domains with non-ribosomal peptide synthases (NRPS), and class IV lanthipeptide clusters (Table 1; Table S5 ). While biosynthetic gene clusters with NRPS domains were only found in *L. jensenii* strains, RiPP-like and class IV lanthipeptide clusters were only found in *L. mulieris* strains.

**TABLE 1:** Number of different BGC types identified for *L. jensenii* and *L. mulieris* genome assemblies examined^*a*^

Species	RiPP-like	NRPS	Lanthipeptide-class-iv	No. of BGCs
*L. jensenii* (*n* = 36)	0	34	0	2
*L. mulieris* (*n* = 25)	3	0	17	5

^*a*^ BGCs, biosynthetic gene clusters; NRPS, non-ribosomal peptide synthases; RiPP, ribosomally synthesized and post-translationally modified peptide.

**Fig 3:**
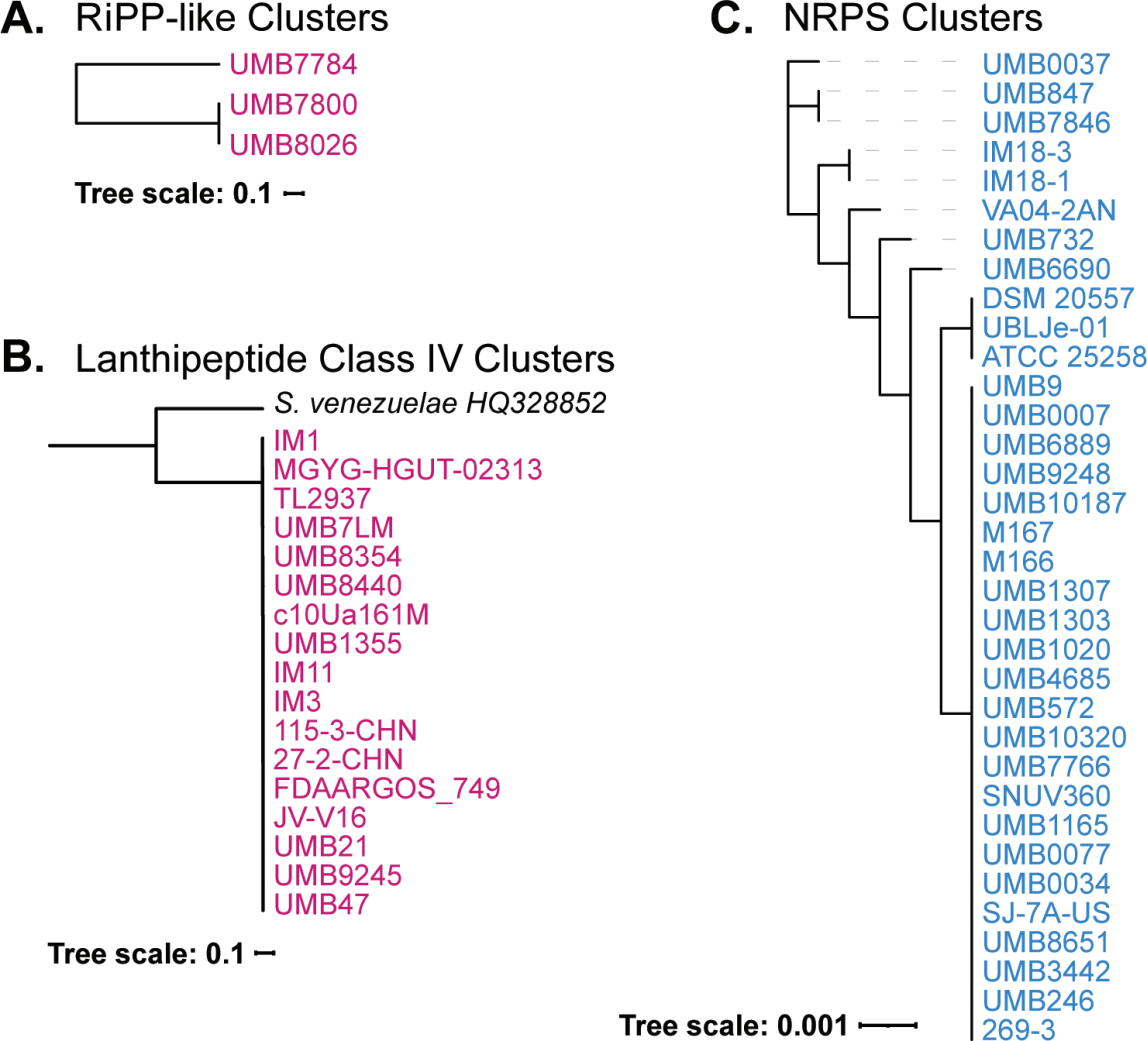
Phylogenetic analysis of BGCs identified in *L. jensenii* (text in blue) and *L. mulieris* (text in pink) strains. (A) *L. mulieris* RiPP-like cluster tree, (B) *L. mulieris* subtree lanthipeptide-class-iv phylogenetic tree, and (C) *L. jensenii* NRPS tree. BGCs, biosynthetic gene clusters; NRPS, non-ribosomal peptide synthases; RiPP, ribosomally synthesized and post-translationally modified peptide.

The RiPP-like BGCs were only found in three genome assemblies: *L. mulieris* strains UMB7784, UMB7800, and UMB8026. These are the three strains that comprise the *L. mulieris* subclade (Fig. 1 and 2). To further investigate the RiPP-like clusters, their nucleotide sequences were aligned, and a phylogenetic tree was derived (Fig. 3A). BAGEL4 analysis identified this RiPP-like BGC as similar to Enterocin NKR-5-3, which was isolated and characterized from *Enterococcus faecium* (36). The RiPP nucleotide sequences also were compared against the NCBI nr/nt database online using blastn. This resulted in homologous hits with a percent identity of 89.9% to *Lactobacillus sakei* IP-TX (accession no. AY206863.1), 88.13% to *E. faecium* (accession no. AB908994.1), and 89.45% to *Latilactobacillus curvatus* (accession no. CP031003.1). This suggests that this BGC may have been acquired from one of these other species. While *L. sakei* and *L. curvatus* are not frequently found in the urogenital tract, *E. faecium* has been identified, most notably in individuals with an infection. In the urinary tract, *E. faecium* is rarely the cause of acute UTIs, but is more frequently associated with catheter-associated UTIs (37). Prior studies also have detected *E. faecium* in the vaginal microbiota of females with bacterial vaginitis (38, 39). Thus, acquisition from *E. faecium* in the urogenital tract is one hypothesis for the acquisition of this BGC. Alternatively, it may have been acquired in the GI tract as *L. curvatus* and *E. faecium* are both members of the human GI microbiota (40, 41). While two *L. mulieris* strains have been isolated from the GI microbiota (Fig. 2), members of the subclade have only been isolated from urine samples. Both *L. curvatus* and *L. sakei* have been explored for their antimicrobial potential (42 - 44), which may be attributed to this BGC. Further research is needed to explore the ability of isolates belonging to the *L. mulieris* subclade’s ability to inhibit/kill uropathogens.

Furthermore, 17 *L*. *mulieris* strains encode for a lanthipeptide-class-iv peptide BGC (Table 1). It is worth noting that the three strains in the *L. mulieris* subclade do not include this lanthipeptide-class-iv peptide. This BGC is found in strains from vaginal, fecal, and urine samples, from healthy and no lower urinary tract symptoms (LUTS) individuals as well as females with LUTS. The representative sequence of the class IV system, venezuelin (accession no. HQ328852) isolated from *Streptomyces venezuelae* (45), was compared to the *L. mulieris* sequences and a phylogenetic tree was derived (Fig. 3B). As displayed in this tree, the *L. mulieris* class IV lanthipeptide nucleotide sequences are essentially identical to each other (99.97% average nucleotide identity) and distinct from venezuelin sequence (29.20% average nucleotide identity). When the venezuelin nucleotide sequence was queried against the NCBI nr/nt database, only hits to *Streptomyces* strains were returned. When the lanthipeptide-class-iv amino acid sequence representative from the complete *L. mulieris* genome strain FDAARGOS_749 (accession no. QGR95320) was queried against the NCBI nr database via blastp, sequence similarity to *L. jensenii/L. mulieris* genomes was identified, as expected. This search also returned high query coverage (>90%) but low percent identity (~38%) to *L. crispatus* protein sequences. A query of the full BGC nucleotide sequence, however, did not identify sequence similarity to any other *Lactobacillus* species. Thus, the *L. mulieris* BGC may represent a new class of lanthipeptide, and further studies are needed to characterize its biological activity.

All but 2 of the *L. jensenii* strains examined here were predicted to contain a BGC containing an NRPS domain. The two *L. jensenii* strains lacking the NRPS domain are UMB0055 and UMB8489. This BGC is found in strains from vaginal, fecal, and urine samples, from healthy and no LUTS individuals as well as females with LUTS. The *L. jensenii* NRPS sequences were aligned, and a phylogenetic tree was derived (Fig. 3C). Upon further analysis, the nucleotide sequence of the NRPS was queried against the NCBI nr/nt database; the only records containing this sequence were from *L. jensenii* strains, suggesting that this sequence is unique to the species. Additional genomes of *Lactobacillus* species commonly found in the female urogenital tract were screened using antiSMASH, finding only one other strain with a predicted NRPS domain, *L. iners* DSM 133335 (accession no. GCF_000160875.1).

### Carbohydrate utilization analysis

**Fig 4:**
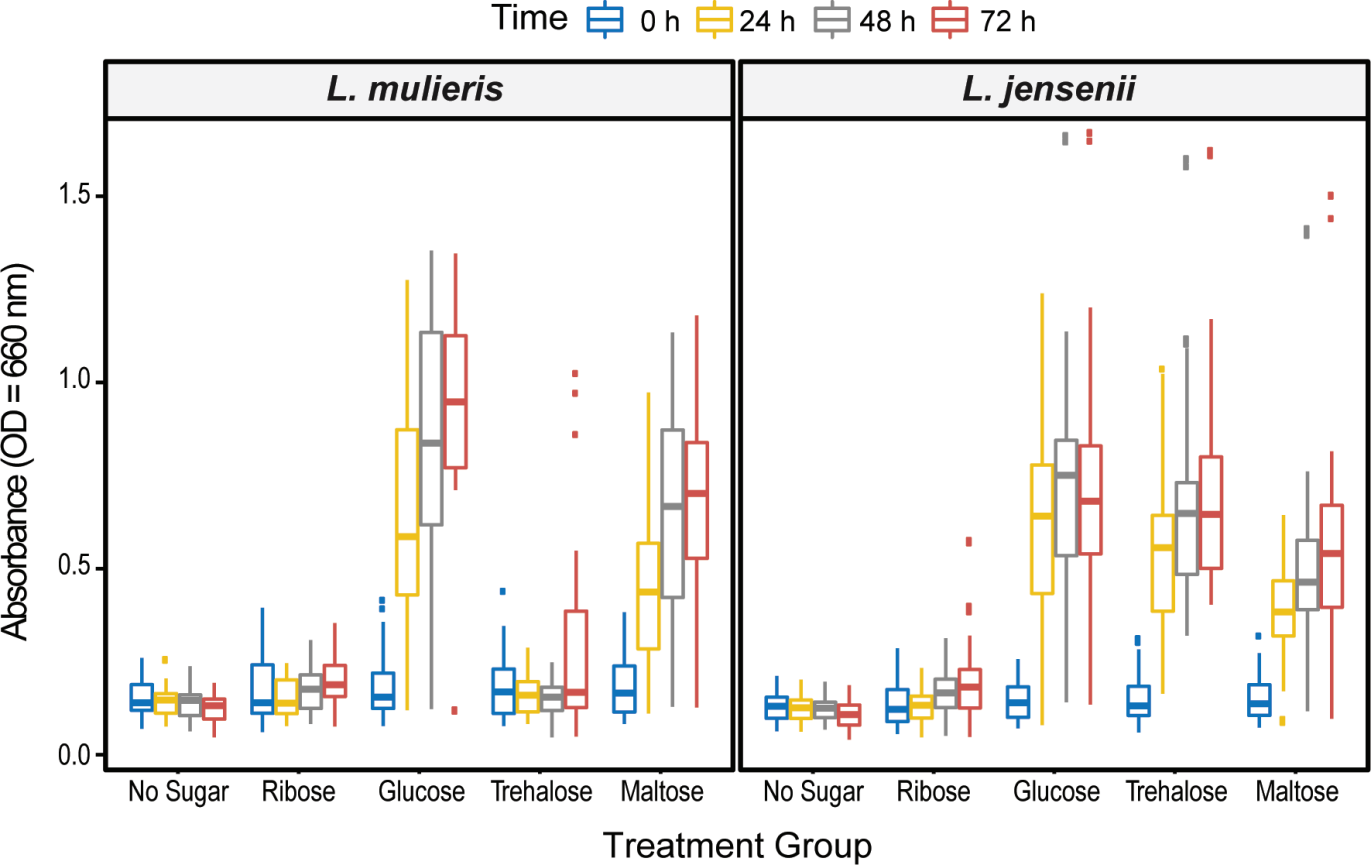
Growth in presence of selected carbohydrates. On the left side, *L. mulieris* is shown, while on the right, *L. jensenii* is shown. The *x*-axis shows each sugar and the *y*-axis shows absorbance. Each different box plot represents the range of growth observed for all strains (and their replicates) under the conditions tested at a single time point. Blue is 0 h, yellow is 24 h, gray is 48 h, and red is 72 h.

Thirty-seven urinary strains from our lab collection of *L. jensenii* (*n* = 24) and *L. mulieris* (*n* = 13) were grown and their ability to utilize four different sugars, ribose, glucose, maltose, and trehalose, was tested alongside media with a no sugar control. These four sugars were selected as in the prior phenotypic characterization of the *L. mulieris* type strain, it was found that API 50 CH results testing ribose and trehalose could distinguish between *L. jensenii* and *L. mulieris* (31); glucose and maltose were selected as both species were expected to catabolize both. Growth was measured at four different time points: 0, 24, 48, and 72 h. Here, we have divided the results for the *L. mulieris* strains (Fig. 4, left panel) and the *L. jensenii* strains (Fig. 4, right panel). As anticipated, no substantial growth was observed for the “no sugar” group (Fig. S2).

As shown in Fig. 4, the *L. mulieris* strains (left panel) were efficient in catabolizing maltose and glucose, but not ribose or trehalose. To determine whether these strains utilized the latter two sugars at all, pairwise comparisons were conducted between them and the “no sugar” (control) group (Table S6). Although there was no statistically significant difference between the control and treatment groups at 0 h and 24 h, at time points 48 h and 72 h, there were statistically significant differences between the no sugar and ribose treatment [*P* = 0.000182 (48 h) and *P* = 1.01 × 10^−08^ (72 h)], and between the no sugar and trehalose treatment [*P* = 0.026 (48 h) and *P* = 1.01 × 10^−08^ (72 h)]. These findings suggest that the *L. mulieris* strains can catabolize ribose and trehalose but not efficiently. In the phenotypic characterization of the *L. mulieris* type strain, the species was characterized by its inability to catabolize ribose and trehalose (31). We find this to be true for <48 h measurements. We conclude that the 13 *L*. *mulieris* strains cannot utilize trehalose or ribose as efficiently as maltose or glucose (Fig. 4).

Our assays suggest that *L. jensenii* strains can efficiently catabolize maltose, trehalose, and glucose, but not ribose. We must note that *L. jensenii* was previously shown to catabolize ribose (31); however, that study assessed just a single strain of *L. jensenii* isolated from the vagina (*L. jensenii* DSM 20557) and no information was provided regarding the culture conditions. We conducted a pairwise comparison of the measurements for *L. jensenii* strains from the control treatment (no sugar) (Table S6) and ribose treatment. At time points 0 h and 24 h, there was no significant difference between the growth of *L. jensenii* strains in the “no sugar” media and the ribose-enriched media. However, a statistically significant difference was detected at time points 48 h and 72 h (*P* = 4.38 × 10^−06^ and 1.10 × 10^−08^, respectively). This suggests that *L. jensenii* can catabolize ribose, but not efficiently. It is worth noting that pairwise comparisons of all sugar treatments to the control treatment for 48 h and 72 h were statistically significant. Nevertheless, in comparison to maltose, trehalose, and glucose, the *L. jensenii* strains cannot utilize ribose nearly as well (Fig. 4).

Fig. 4 also includes outliers to these general trends. For *L. mulieris*, there are outliers for trehalose at 0 h and 72 h. At time point 0 h, the outlier is one of the three biological replicates for UMB0047, which comes from a no LUTS (asymptomatic) participant. However, measurements for this line at subsequent time points did not deviate from the measurements from other strains. At time point 72 h, the three outlier points are the three biological replicates of UMB4707, a clinical isolated from an OAB+ patient. The increased growth of this strain at 72 h suggests that this strain can catabolize trehalose. These outliers may impact the statistical significance identified for the 72 h trehalose data.

Outliers also were observed for the *L. jensenii* strains for growth supplemented with maltose, trehalose, and glucose; these include the highest growth rates observed. The outliers of *L. jensenii* for maltose time point 0 h are the biological replicates of UMB0055, a strain from a female with no LUTS. At time points 48 h and 72 h, the outliers are the three replicates of UMB1303, a clinical isolated from a participant with a clinical diagnosis of an acute UTI. While maltose is present in low concentrations in urine, the vaginal microbiota can degrade α-amylase activity and breakdown glycogen into maltose due to the presence of *Lactobacillus* species (11, 46, 47). Other outliers of *L. jensenii* can be found in the trehalose treatment at 48 h and 72 h. These outliers are again UMB1303 at both time points. Upon further inspection of UMB1303, it grew best in all carbohydrate conditions, including ribose and glucose, at all time points.

**Fig 5:**
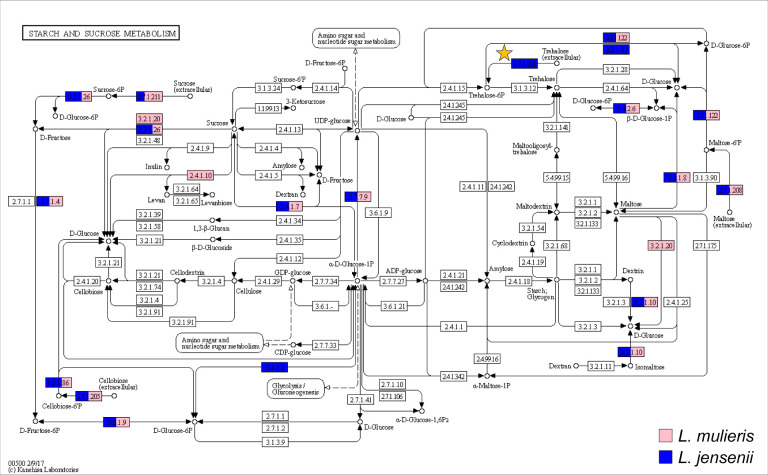
Metabolic pathway of starch and sucrose metabolism for *L. jensenii* and *L. mulieris. L. jensenii* presence is shown in blue and *L. mulieris* presence is shown in pink. The white boxes mean that those elements are missing. EC2.7.1.201 (trehalose-specific PTS system EIIA component, TreB), which is required for trehalose-specific transport of extracellular trehalose, is indicated by the yellow star.

Based upon the assays performed here, the ability to utilize trehalose is a distinguishing characteristic between these two species. To further investigate this observation, the genome sequences for the two type strains, *L. jensenii* SNUV360 (accession no. CP018809.1), which was isolated from the vagina, and *L. mulieris* strain c10Ua161M (accession no. GCA_007095465.1), were examined. Specifically, we examined the gene content identified for the KEGG starch and sucrose metabolism pathway; this pathway was selected as it includes catabolism and transport of trehalose, among other sugars tested here. As shown in Fig. 5, only the *L. jensenii* strain encodes for the EC2.7.1.201 (trehalose-specific phosphotransferase system [PTS] enzyme IIA [EIIA] component, TreB) (Fig. 5, yellow star), which is required for trehalose-specific transport of extracellular trehalose (as indicated in blue). *L. mulieris* UMB4707, in which all three biological replicates were outliers in the 72 h measurements (although not prior) for the trehalose treatment, does not encode for EC2.7.1.201. Thus, it is unclear how this strain had increased growth at 72 h relative to other *L. mulieris* strains.

The ability to catabolize the sugars tested can provide insight into the species’ persistence in the female urogenital tract. Glucose and maltose are common byproducts of glycogen which accumulates in the vaginal epithelial cells starting at puberty (48). Thus, it is not surprising that both species are able to catabolize both glucose and maltose efficiently. In *Lactobacillus acidophilus*, glycogen metabolism was found to be repressed by glucose, but it was at the highest intracellular levels in the presence of trehalose (49). Prior studies of lactobacilli of the vagina noted that *L. crispatus* and *L. iners* also encode for the trehalose PTS gene and can metabolize trehalose (50, 51). Variation has been observed for another urogenital *Lactobacillus* species, *L. gasseri* (51). A prior study found that bacteriocin production was enhanced when a *L. sakei* culture was supplemented with trehalose (52), suggesting future studies of *L. jensenii* BGC production in the presence/absence of trehalose. No other PTS system genes were present in *L. mulieris* strains and were absent in *L. jensenii* strains, signifying that *L. mulieris* has limited resources for glycolysis in comparison to other urogenital lactobacilli. Further investigation into the ramifications for this observation is needed.

## MATERIALS AND METHODS

### Data acquisition

The publicly available genome sequences of *L. jensenii* and *L. mulieris,* totaling 45 strains were retrieved from NCBI’s RefSeq database as of 18 September 2021. Metagenomic assembled genomes were not included in our analysis. CheckM was used to verify that the assemblies were complete (>96%) and had minimal contamination (<5%) (53). Nine additional urinary strains in our collection were sequenced as described in subsequent sections, and their genomes were deposited in NCBI and included in our analysis. Table S1 lists the sequences included in this study and their information, including the strain name, isolation source, strain, length, number of contigs, and GC%. For those strains in which metadata is listed for the symptom status of the individual, this is also included in Table S1.

### Sample acquisition

*L. jensenii* and *L. mulieris* strains tested were obtained through prior Institutional Review Board (IRB)-approved studies (IRB approvals LUC206469, LUC207102, and LUC204195 from Loyola University Chicago and 17077AW from University of California San Diego) (3, 18, 54 - 56). Briefly, catheterized samples were collected and cultured using the Expanded Quantitative Urinary Culture (56). Strains were identified as *L. jensenii* by matrix-assisted laser desorption/ionization-time of flight (MALDI-TOF) mass spectrometry [as previously described (18)] and stored at −80°C. All samples were isolated and identified via MALDI-TOF prior to the description of *L. mulieris*. Freezer stocks were first streaked on Columbia colistin naladixic acid (CNA) agar with 5% sheep blood plates (BD 221353) and incubated at 35°C in 5% CO_2_ for 48 h. Next, a single colony was selected and grown in De Man, Rogosa, and Sharpe (MRS) broth (Sigma-Aldrich) supplemented with 1% Tween 80 at 35°C in 5% CO_2_ for 48 h. This culture was then stored in 50/50 v/v glycerol at −80°C. Strains from our own collection (indicated by the prefix “UMB”) are available from the authors upon request.

### Sequencing urinary isolates

The following protocol was used to generate the nine genomes produced as part of this work. The methods employed mirror those used to produce seven genomes recently reported in the literature (57). Briefly, samples were extracted using a modified version of the Qiagen Blood and Tissue Kit Protocol (see reference for modifications). Samples were sequenced at MIGS (Pittsburgh, PA). There, sequencing libraries were prepared using the Illumina Nextera Kit, and samples were sequenced using the Illumina NextSeq 550 platform (150 bp × 2, next spaired-end reads). Raw reads were first trimmed for quality using BBDuk v. 38.92 (https://sourceforge.net/projects/bbmap/) with the following parameters: “*ftl = 15, ftr = 135, minlength = 30, qtrim = rl, maq = 20, maxns = 0, statscolumns = 5, trimq = 20*.” The genomes were assembled via SPAdes v. 3.15.2 using the assembly-only option (58). Genome assemblies were made publicly available by depositing them in NCBI’s Assembly database. When deposited, the genome assemblies were annotated using the NCBI Prokaryotic Genome Annotation Pipeline (PGAP) v. 5.3 (59).

### Bioinformatic analysis

The 16S rRNA reference sequence for *L. jensenii* was obtained from the SILVA database (60). This sequence was used to create a local nucleotide blast database (61). Each genome was then queried against this database using blastn to find the 16S rRNA sequence. The resulting 16S rRNA gene sequences were imported into Geneious Prime (Biomatters Ltd., Auckland, NZ) and aligned using the MAFFT v7.388 (62) plug-in through Geneious Prime. Nucleotide differences between the sequences were identified, and their location within conserved or variable regions was determined by aligning the consensus sequence against the *E. coli* 16S rRNA gene sequence (GenBank accession no. J01859.1) (63).

The average nucleotide identity (ANI) was computed using pyani v0.2 (64). From the ANI percentage identity values, we classified the genomes into species based on the 95% ANI threshold routinely used in the field for distinguishing between closely related bacterial taxa (33).

Each genome was screened for secondary metabolites via antiSMASH using the default parameters and all extra features option of the web-based tool (65). The biosynthetic gene clusters sequences found by antiSMASH were aligned using MAFFT (v7.388) (62) and a phylogenetic tree was created as described below. Genomes also were screened using BAGEL4 through the web interface (66).

The core for the publicly available genomes and genomes sequenced as part of this work were determined using anvi’o v7.1 (67). First, contigs less than 1,000 bp were removed using the command *anvi-script-reformat-fasta*; afterward, the command *anvi-gen-contigs-database* was used to generate databases for each genome. The *anvi-pan-genome* command was used to create the pangenome of all the genomes with an “*MCL-inflation*” parameter value of 9. Our previous analysis included an MCL-inflation parameter value of 10, assuming they are the same species, but because we know they are two different species, we decreased this value to 9 (32). The concatenated single-copy core genome was found using the command *anvi-get-sequences-for-gene-clusters* with the following parameters: *“--min-num-genomes-gene-cluster-occurs number_of_genomes --max-num-genes-from-each-genome 1 --concatenate-gene-clusters,*” where the variable *number_of_genomes* is equivalent to the number of genomes in the data set. The output for this command is amino acid sequences.

Phylogenies of sequences were created as follows. Sequences were imported into Geneious Prime 2022.1.1 (Biomatters Ltd., Auckland, NZ) and aligned using the MAFFT v7.388 (62) plug-in through Geneious Prime. Phylogenetic trees were derived using the FastTree 2.1.12 (68) plug-in through Geneious Prime and visualized using iTOL v6 (69).

The type strain for *L. jensenii* and *L. mulieris* was annotated using RAST (70) and their EC numbers were extracted. The KEGG Color mapper was used to annotate their carbohydrate pathway source from the RAST annotation (71).

### Carbohydrate utilization assays

*Lactobacillus* isolates from our collection were streaked onto MRS + 1% Tween 80 agar plates from freezer stocks and incubated overnight at 35°C with 5% CO_2_. Colonies were picked from these plates to inoculate 10 mL of MRS + 1% Tween 80 liquid media and grown for 48 h at 35°C and 5% CO_2_. This was performed in triplicate for each strain. After 48 h, each bacterial culture was pelleted by centrifugation (7,000 rpm for 10 min). The spent media were removed, and the pellet was washed with 10 mL of phosphate-buffered saline (PBS). The pellet was washed again before resuspension in 15 mL semi-defined basal medium (SDM) without any carbohydrates [Tween 80: 1 g, ammonium citrate: 2 g, sodium acetate buffer solution (pH 5.2 ± 1 at 25°C): 20.316 mL, MgSO_4_ · 7 H_2_O: 0.1 g, MnSO_4_: 0.05 g, K_2_HPO_4_: 2 g, yeast nitrogen base (Diftco): 5 g, and Bacto Casiton: 10 g in 1 L H_2_O]. This recipe was adapted from reference (72). The triplicate samples for each strain were then combined into 50 mL conical tubes and mixed by vortexing for 30 s. 1 mL of the vortexed culture was added to 19 mL of SDM media supplemented with 20 g/1 L of one of the four sugars: (i) trehalose dihydrate (VWR), (ii) D-(-)-ribose, 98% (BeanTown Chemical), (iii) maltose monohydrate (VWR), or (iv) d-glucose (Dextrose) (TEKnova). SDM media with no supplementation were used to serve as a control. Each strain was tested with three biological replicates.

For each of these cultures, bacterial growth was measured using a spectrophotometer (wavelength = 660 nm) at 0 h, 24 h, 48 h, and 72 h. The spectrophotometer was calibrated for each of the media using the SDM media + sugar (or no sugar in the case of the control) without bacteria. At each time point, 1 mL was removed from the culture for measurement. All measurements were recorded for statistical analyses.

For each time point, *L. jensenii* and *L. mulieris* strain measurements, which were conducted in triplicate (biological replicates), were considered independent replicates of their respective species. While this was mainly done for convenience, we also verified statistically that there was no significant difference between the different strains of a species. Exploratory data analysis in R was used to visualize our data. We then performed a repeated measures analysis of variance (ANOVA) (73) where the factors were species (two levels: *L. mulieris*, *L. jensenii*) and sugar (five levels: each sugar and the no sugar control), over the four-time points (0 h, 24 h, 48 h, and 72 h). This was followed by two-way ANOVA models in R to identify other significant differences. Pairwise comparisons were performed at each time point for each species to identify significant differences between treatments. To control the family wise error rate of such comparisons, a Bonferroni correction was used.

## SUPPLEMENTAL MATERIAL

Fig. s1 msphere.00562-22-s0001.tif Phylogenetic tree of the 16S rRNA gene sequences.

Fig. s2 msphere.00562-22-s0002.tif Absorbance with 95% confidence intervals for each treatment group over time with *L*. *mulieris* strains in the left panel and *L. jensenii* strains in the right panel.

Table S1 msphere.00562-22-s0003.xlsx Genome information for *L*. *jensenii* and *L*. *mulieris* strains examined.

Table S2 msphere.00562-22-s0004.xlsx Pairwise ANI values for *L. jensenii L. mulieris* genomes examined.

Table S3 msphere.00562-22-s0005.docx *L. mulieris*-specific COG functions.

Table S4 msphere.00562-22-s0006.docx *L*. *jensenii*-specific COG functions.

Table S5 msphere.00562-22-s0007.xlsx Strains containing BGCs.

Table S6 msphere.00562-22-s0008.xlsx Pairwise statistical comparison of sugar treatments to the “No Sugar” control treatments over time.

ASM does not own the copyrights to Supplemental Material that may be linked to, or accessed through, an article. The authors have granted ASM a non-exclusive, world-wide license to publish the Supplemental Material files. Please contact the corresponding author directly for reuse.
