# Supplementary figures and images for: Exploring the genotypic and phenotypic differences distinguishing Lactobacillus jensenii and Lactobacillus mulieris
Source: mSphere. 2023 Jun 27;8(4):e00562-22. doi: 10.1128/msphere.00562-22 (PMC10449518; doi:10.1128/msphere.00562-22)

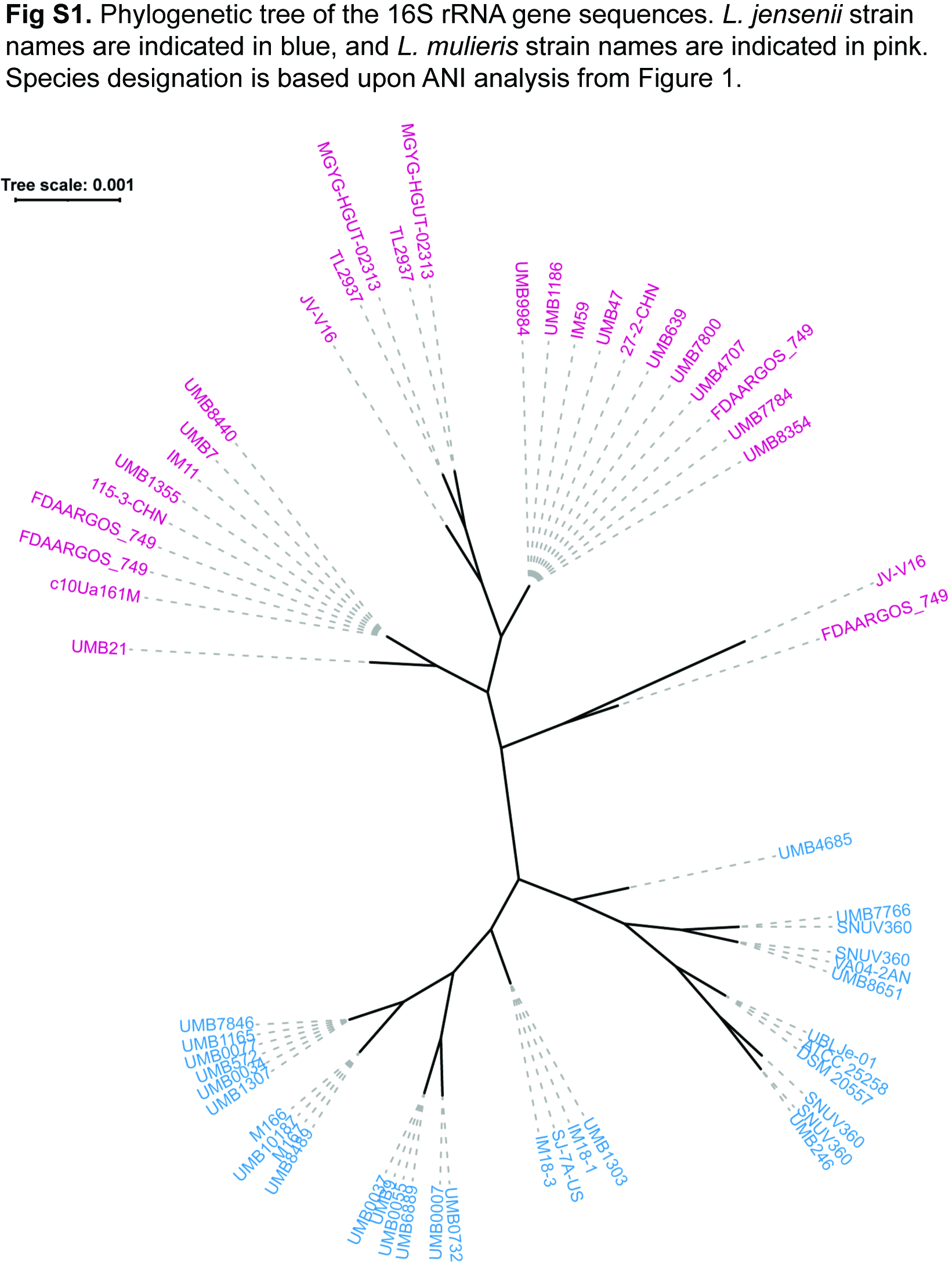

Supplement: Fig. s1 — Phylogenetic tree of the 16S rRNA gene sequences. [file msphere.00562-22-s0001.tif]

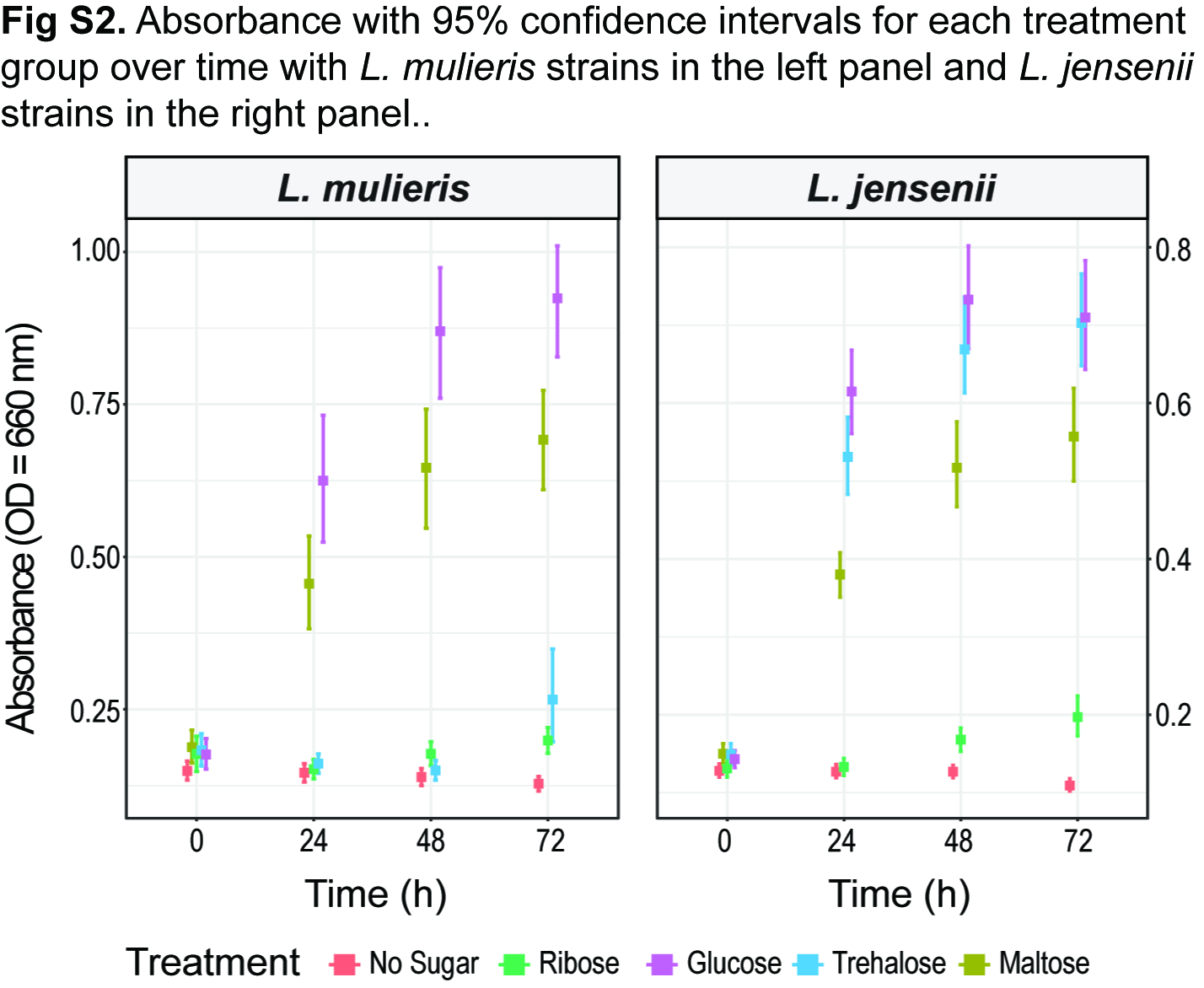

Supplement: Fig. s2 — Absorbance with 95% confidence intervals for each treatment group over time with L. mulieris strains in the left panel and L. jensenii strains in the right panel. [file msphere.00562-22-s0002.tif]
