# Supplementary material for: Exploring the genotypic and phenotypic differences distinguishing Lactobacillus jensenii and Lactobacillus mulieris
Source: mSphere. 2023 Jun 27;8(4):e00562-22. doi: 10.1128/msphere.00562-22 (PMC10449518; doi:10.1128/msphere.00562-22)
Supplement: Table S3 — L. mulieris-specific COG functions. [file msphere.00562-22-s0005.docx]

**Table S3.** *L. mulieris*-specific COG functions

| **COG category** | **COG20_FUNCTION** | **COG ID** |
| --- | --- | --- |
| Nucleotide transport and metabolism | Cytosine/adenosine deaminase or related metal-dependent hydrolase (SsnA) (PDB:3O7U) | COG0402 |
| Cell cycle control and mitosis | Zn-dependent membrane protease YugP (YugP) | COG2340, COG5271 |
| Amino Acid metabolism and transport | 5-carboxyvanillate decarboxylase LigW (lignin degradation), amidohydro domain (LigW) (PDB:2DVT) (PUBMED:26714575) | COG1168 |
| Carbohydrate metabolism and transport | NADPH-dependent 2,4-dienoyl-CoA reductase, sulfur reductase, or a related oxidoreductase (FadH2) (PDB:1VRQ) | COG2159 |
| Carbohydrate metabolism and transport | Uncharacterized conserved protein YxeA, DUF1093 family (YxeA) (PDB:2K5Q) | COG3507 |
| Carbohydrate metabolism and transport | Beta-xylosidase (XynB2) (PDB:1Y7B) | COG1196, COG1501 |
| Carbohydrate metabolism and transport and Amino Acid metabolism and transport | Chromosome segregation ATPase Smc (Smc) (PDB:5XG3); Alpha-glucosidase/xylosidase, GH31 family (YicI) (PDB:1WE5) | COG1363 |
| Coenzyme metabolism | Protoheme ferro-lyase (ferrochelatase) (HemH) (PDB:1AK1) | COG0276 |
| Coenzyme metabolism | Uncharacterized conserved protein, DUF1430 domain | COG0667 |
| Lipid metabolism | Pyridoxal reductase PdxI or related oxidoreductase, aldo/keto reductase family (PdxI) (PDB:1LQA) | COG0446 |
| Translation | Putative aminopeptidase FrvX (FrvX) (PDB:1VHE) | COG5271 |
| Post-translational modification, protein turnover, chaperone functions | Ammonia channel protein AmtB (AmtB) (PDB:1U77) | COG2738 |
| Inorganic ion transport and metabolism | Midasin, AAA ATPase with vWA domain, involved in ribosome maturation (MDN1) (PDB:6HYP) | COG0004 |
| Function Unknown | Bifunctional PLP-dependent enzyme with beta-cystathionase and maltose regulon repressor activities (MalY) (PDB:4DGT) | COG5294 |
| Function Unknown | Spore germination protein YkwD and related proteins with CAP (CSP/antigen 5/PR1) domain (YkwD) (PDB:1CFE) (PUBMED:31199835)!!!Midasin, AAA ATPase with vWA domain, involved in ribosome maturation (MDN1) (PDB:6HYP) | COG4652 |
| Function Unknown | Uncharacterized conserved protein, DUF2316 domain | COG4367 |
