# Supplementary material for: Exploring the genotypic and phenotypic differences distinguishing Lactobacillus jensenii and Lactobacillus mulieris
Source: mSphere. 2023 Jun 27;8(4):e00562-22. doi: 10.1128/msphere.00562-22 (PMC10449518; doi:10.1128/msphere.00562-22)
Supplement: Table S4 — L. jensenii-specific COG functions. [file msphere.00562-22-s0006.docx]

**Table S4.** *L. jensenii*-specific COG functions

| **COG category** | **COG20_FUNCTION** | **COG ID** |
| --- | --- | --- |
| Energy production and conversion | FAD/FMN-containing lactate dehydrogenase/glycolate oxidase (GlcD) (PDB:1AHU) | COG1154 |
| Energy production and conversion | Cytochrome bd-type quinol oxidase, subunit 2 (AppB) (PDB:6RKO) | COG4988 |
| Energy production and conversion | NADH dehydrogenase, FAD-containing subunit (Ndh) (PDB:5NA4) | COG0277 |
| Energy production and conversion | Cytochrome bd-type quinol oxidase, subunit 1 (AppC) (PDB:5DOQ) | COG1294 |
| Carbohydrate metabolism and transport | Glycogen debranching enzyme (alpha-1,6-glucosidase) (GDB1) (PDB:5D06) | COG1575 |
| Carbohydrate metabolism and transport | Glucose uptake protein GlcU (GlcU) | COG4987 |
| Coenzyme metabolism | 1,4-dihydroxy-2-naphthoate polyprenyltransferase (MenA) | COG1252 |
| Coenzyme metabolism and Lipid metabolism | Deoxyxylulose-5-phosphate synthase (Dxs) (PDB:2O1S) | COG3408 |
| Post-translational modification, protein turnover, chaperone functions | ABC-type transport system involved in cytochrome bd biosynthesis, ATPase and permease components (CydD) | COG4975 |
| Post-translational modification, protein turnover, chaperone functions | ABC-type transport system involved in cytochrome bd biosynthesis, fused ATPase and permease components (CydC) | COG1271 |
| Secondary Structure | Acyl carrier protein (AcpP) (PDB:1ACP)!!!EntF, seryl-AMP synthase component of non-ribosomal peptide synthetase (EntF) (PDB:5ES5) | COG0236, COG1020 |
